# Development and Preliminary Psychometric Evaluation of a Melanoma Risk, Knowledge and Protective Behavior Questionnaire for Serbian Patients

**DOI:** 10.3390/healthcare14172822

**Published:** 2026-09-03

**Authors:** Vladimir Vidović, Jelena Radić, Ivana Kolarov Bjelobrk, Ivana Davidov, Mihajlo Erdeljan, Annamaria Galfi Vukomanović, Bojana Blagojević

**Affiliations:** 1 Department of Medical Oncology, Oncology Institute of Vojvodina, Put doktora Goldmana 4, 21204 Novi Sad, Serbia; vladimir_vidovic@hotmail.com (V.V.); jelena.radic@mf.uns.ac.rs (J.R.); ivana.kolarov-bjelobrk@mf.uns.ac.rs (I.K.B.); 2 Department of Veterinary Medicine, Faculty of Agriculture, University of Novi Sad, Trg Dositeja Obradovića 8, 21000 Novi Sad, Serbia; ivana.davidov@polj.edu.rs (I.D.); annamariagalfi@gmail.com (A.G.V.)

**Keywords:** melanoma, questionnaire validation, psychometric evaluation, melanoma knowledge, melanoma risk perception, sun-protective behavior, prevention, Serbia

## Abstract

**Background/Objectives:** Effective melanoma prevention depends on reliable instruments capable of assessing individual risk factors, knowledge, attitudes, risk perception, and preventive behaviors. However, no culturally adapted questionnaire has been available for Serbian melanoma patients. This study aimed to develop and evaluate the psychometric properties of a Serbian questionnaire designed to assess melanoma-related risk factors, knowledge, concern, attitudes, and sun-protective behavior in a clinical population. **Methods:** A cross-sectional study was conducted among 333 melanoma patients treated at the Oncology Institute of Vojvodina, Serbia. The questionnaire was developed using the conceptual framework of a previously published instrument as a methodological reference and was modified and expanded for the Serbian clinical setting. Internal consistency was evaluated using Cronbach’s alpha. Differences between participant groups were analyzed using independent-samples *t*-tests and one-way ANOVA. Correlations between questionnaire domains were assessed using Spearman’s correlation coefficients, while predictors of sun-protective behavior were identified using multivariable linear regression. **Results:** The developed questionnaire demonstrated satisfactory internal consistency, with Cronbach’s alpha coefficients ranging from 0.711 to 0.874. Participants demonstrated high levels of melanoma-related knowledge and attitudes, melanoma-related concern, and reported sun-protective behavior, whereas perceived personal melanoma risk was comparatively lower. Female participants reported significantly greater melanoma-related concern and more frequent sun-protective behavior than males (*p* < 0.05). Significant positive correlations were observed among all questionnaire domains, with the strongest associations identified between melanoma-related concern and sun-protective behavior and between knowledge and sun-protective behavior (both *p* < 0.001). Multivariable analysis demonstrated that melanoma-related concern and knowledge independently predicted adequate sun-protective behavior, explaining 61.2% of its variance. **Conclusions:** The developed Serbian questionnaire demonstrated satisfactory psychometric performance and represents a practical instrument for assessing melanoma-related knowledge, perceptions, and preventive behaviors in clinical settings. The findings emphasize the importance of combining knowledge with behavioral motivation and support the implementation of targeted educational interventions to improve melanoma prevention among high-risk individuals.

## 1. Introduction

Cutaneous melanoma (CM) is the type of skin cancer associated with the highest burden of morbidity, mortality, and years of potential life lost [1,2,3]. However, CM can be largely prevented through patient education, sun protection, screening, and public health campaigns [4,5,6,7]. These comprehensive initiatives have included multimedia campaigns, educational programs, organizational and environmental strategies, and policies that have led to well-documented improvements in sun protective behaviors [4,5,6,8]. The positive impact of these programs was further evidenced by a plateau in CM incidence in the mid-1990s and a decline in incidence among individuals aged 15 to 24 years [1,8]. A critical factor in the success of these campaigns was the integration of research and evaluation throughout their planning, implementation, and development phases [8]. Recent evidence highlights the importance of tailoring public health messages to specific population groups and using evidence-based communication strategies to improve melanoma awareness, skin-check intentions, and adherence to preventive behaviors [7].

Globally, the incidence rates of CM have been rising over recent decades, becoming a public health concern worldwide, including in North America [9,10,11]. For example, studies from Canada have demonstrated a rise in incidence rates from 12.29 per 100,000 individuals per year during 1992–2010 to 20.75 during 2011–2017, with significant regional differences within the country [12,13]. Recent population-based data from the United States further demonstrate that, despite overall declines in age-standardized melanoma incidence and mortality, substantial disparities persist according to sex, age, geographic location, and sociodemographic characteristics [14]. Importantly, recent comprehensive reviews emphasize that such trends are influenced by a complex interplay of genetic, environmental, and behavioral factors, necessitating multidimensional approaches to prevention [15].

Interestingly, melanoma is not a disease exclusive to humans; it also occurs spontaneously in several animal species, particularly in dogs, horses, and cats. Comparative oncology studies have shown that certain biological and molecular characteristics of melanoma, including patterns of tumor progression and metastatic behavior, share similarities across species. In a comparative epidemiologic analysis, the incidence rate of melanocytic tumors was reported at approximately 16.1 per 100,000 dogs per year, compared with 8.1 per 100,000 humans and 6.3 per 100,000 cats, underscoring species-specific differences in frequency and presentation [16]. These parallels have contributed to a broader understanding of melanoma pathogenesis and have supported the development of translational research models. Nevertheless, despite these biological similarities, the primary drivers of melanoma burden in humans remain strongly linked to modifiable environmental and behavioral factors, particularly ultraviolet radiation exposure. Therefore, while insights from veterinary oncology enrich the biological understanding of melanoma, public health strategies in humans continue to focus predominantly on prevention, early detection, and behavior change.

In Serbia, melanoma is considered less common compared to Western European countries, but recent epidemiological data indicate a worrying increase in melanoma mortality rates, particularly among specific age and sex groups. Population-based studies have shown an average annual increase in melanoma mortality of approximately 1.43% between 2000 and 2021, with significantly higher growth rates observed among men aged 65–69 and women aged 35–39 [17,18,19,20]. This trend highlights an urgent need for targeted melanoma prevention and early detection strategies in Serbia. In this context, the increasing melanoma mortality burden further emphasizes the need for culturally appropriate and systematically evaluated instruments capable of assessing melanoma-related risk factors, knowledge, risk perception, and preventive behaviors, which may support the identification of patients requiring targeted preventive interventions.

Currently, Serbia lacks coordinated, long-term preventive initiatives. Existing efforts have primarily focused on diagnosis and treatment rather than primary prevention. The absence of widespread, evaluated prevention programs means that melanoma incidence and mortality continue to rise, underscoring the need for effective tools to identify those at greatest risk and to deliver targeted prevention advice.

Identifying individuals at highest risk of developing melanoma, as well as assessing their knowledge and protective behavior, is crucial for developing effective prevention measures. Questionnaires that reliably measure these factors can be valuable tools in clinical and public health settings [21,22,23]. As Smit et al. [22] pointed out, the development and validation of self-assessment questionnaires improve patient-physician communication, enabling personalized risk communication and better adherence to preventive behaviors. Furthermore, Smit et al. (2024) [23] demonstrated that structured risk assessment tools, combined with patient education, significantly enhance early detection and improve sun protection compliance. However, such instruments must be culturally adapted and validated to ensure their relevance and accuracy in the Serbian population [19,24].

Moreover, recent research highlights the importance of integrating intrinsic and extrinsic risk factors into comprehensive melanoma risk assessments [15,25]. These tools, supported by evidence-based prevention strategies, have become even more critical in the evolving landscape of melanoma prevention and control [18]. According to Usher-Smith et al. [15], comprehensive risk models that combine genetic predisposition, phenotypic traits, and sun exposure history allow for more precise identification of high-risk individuals, enabling more targeted interventions. Such findings emphasize that instruments measuring melanoma-related risk, knowledge, protective behavior, and attitudes must be culturally adapted and validated to ensure relevance and accuracy within specific populations, including Serbia.

From a clinical perspective, such an instrument may help identify patients with insufficient melanoma-related knowledge, low perceived personal risk, or inadequate sun-protective behavior, thereby allowing healthcare professionals to provide more individualized counselling and preventive education. At the population level, the identification of demographic and behavioral subgroups with lower preventive engagement may also support the development of targeted melanoma prevention strategies in Serbia.

The aim of this study was to develop and preliminarily evaluate a questionnaire designed to assess melanoma-related risk factors, knowledge, attitudes, concern, and protective behavior among patients attending the Oncology Institute of Vojvodina. In addition, the study aimed to examine the reliability of the questionnaire and to identify demographic and clinical factors associated with melanoma-related perceptions and sun-protective behavior in the Serbian population.

## 2. Materials and Methods

### 2.1. Setting

This study was conducted at the Oncology Institute of Vojvodina in Sremska Kamenica, Serbia. Participants included patients diagnosed with melanoma who were undergoing treatment and follow-up care at this institution.

The questionnaire was developed by the research team based on previously published instruments assessing melanoma risk, knowledge, attitudes, and protective behavior. The wording of individual items was modified to ensure suitability for the Serbian population and the clinical setting of the Institute of Oncology of Vojvodina.

The questionnaire was developed by the research team using the instrument originally reported by Gillespie et al. [20] as a methodological reference and was subsequently modified and expanded for use in the Serbian clinical setting. The present study represents a preliminary evaluation of its measurement properties and practical applicability in a clinical population.

### 2.2. Ethical Approval

Full ethical approval for this study was granted by the Ethics Committee of the Oncology Institute of Vojvodina, Sremska Kamenica, Serbia (Reference number: 4/25/3-4367/2-8), prior to the commencement of data collection. The study protocol and questionnaire were reviewed and approved in accordance with the principles for research involving human participants. All participants provided informed consent before completing the questionnaire, and participation was voluntary and anonymous.

### 2.3. Participants

The questionnaire was completed in a written format by a convenience sample of patients diagnosed with melanoma who were currently undergoing treatment and follow-up care at the Oncology Institute in Sremska Kamenica. Eligible participants were all patients with a confirmed diagnosis of melanoma who were either undergoing treatment or attending follow-up visits at the Oncology Institute of Vojvodina. Only patients meeting these criteria were invited to participate. All invited participants agreed to complete the questionnaire, and no eligible patient refused participation. A total of 333 participants completed the questionnaire, and all 333 questionnaires were included in the final analysis. Missing responses to individual questionnaire items were very limited and were treated as missing; no imputation was performed. One participant did not complete the 17 Likert-scale items; therefore, composite domain scores could not be calculated for this participant, who was excluded only from analyses involving the four Likert-scale domain scores. Consequently, these analyses were based on 332 participants. Given the very small proportion of missing responses, this approach was considered to have a negligible impact on the results while allowing all available observations to be retained in the analysis. Participants were aged between 30 and 80 years. For analytical purposes, participants were categorized into three age groups (≤45 years, 46–65 years, and ≥66 years). The categorization was based on previously reported age-specific differences in melanoma incidence, mortality, and preventive behavior, particularly among middle-aged and older adults, as reported in previous Serbian epidemiological studies [17,18]. This approach enabled the assessment of potential age-related differences in melanoma-related knowledge, concern, and protective behavior. The questionnaire was adapted to include additional demographic variables as well as detailed questions on UV exposure, sun protection practices, melanoma knowledge, and level of concern about melanoma. This version of the questionnaire was subsequently reviewed by the research team to ensure clarity and suitability for the study population [21].

### 2.4. Questionnaire and Data Collection

The questionnaire was developed by the research team using the conceptual framework and domain structure reported by Gillespie et al. [21] as a methodological reference. The Gillespie questionnaire comprises demographic items followed by four domains assessing personal melanoma risk, level of concern, protective behaviour, and melanoma knowledge. These domains were retained as the conceptual basis of the present instrument; however, the individual items and response options were modified and expanded to reflect the Serbian clinical setting and the characteristics of patients with a confirmed melanoma diagnosis. As the questionnaire was not intended to represent a direct Serbian translation of the original Gillespie et al. instrument, a formal forward–backward translation procedure was not performed. Consequently, the present study should be regarded as a preliminary evaluation of a newly developed, context-specific questionnaire rather than as a formal linguistic validation of a translated instrument.

The resulting questionnaire consisted of 47 items. The first 30 items included six demographic and melanoma-history questions, seven items assessing personal melanoma risk, four items assessing melanoma-related concern and self-examination behaviour, seven items assessing sun-protective behaviour, and six items assessing melanoma knowledge. In addition, 17 Likert-scale statements were developed to provide a more detailed assessment of perceived personal risk, concern, protective behaviour, and melanoma-related knowledge and attitudes. These statements were rated on a five-point scale ranging from 1 (“strongly disagree/never”) to 5 (“strongly agree/always”).

The first six items collected demographic data and information on participants’ personal and family history of melanoma. The following sections included multiple-choice items grouped into key domains: personal risk factors for melanoma (phenotypic characteristics and sun exposure habits), level of concern and self-examination behaviour, sun protection practices, and knowledge about melanoma. These sections comprised 30 structured questions with predefined response options.

In addition, the final section of the questionnaire consisted of 17 statements assessing perceived personal risk, level of concern, knowledge, attitudes, and protective behaviour related to melanoma. Consistent with the original questionnaire developed by Gillespie et al. [21], these statements were rated using a five-point Likert scale a widely used approach for measuring attitudes and perceptions in questionnaire-based research, ranging from 1 (“strongly disagree/never”) to 5 (“strongly agree/always”) [26].

The questionnaire included both categorical items and Likert-scale items. The categorical items describing demographic characteristics, melanoma history, risk factors, knowledge, and protective behaviors were analyzed descriptively and were not included in the calculation of the four composite domain scores. The 17 Likert-scale items were grouped into four conceptual domains: perceived personal melanoma risk, melanoma-related concern, sun-protective behavior, and knowledge and attitudes toward melanoma. Responses to the Likert-scale items within each domain were averaged to generate the corresponding composite domain score. Because internal consistency is meaningful for multi-item scales, reliability analysis using Cronbach’s alpha was performed only for these four Likert-scale domains.

### 2.5. Statistical Analysis

Statistical analysis was performed using R version 4.6.0. (R Foundation for Statistical Computing, Vienna, Austria) [27]. Before analysis, the dataset was checked for missing values, coding errors, and inconsistencies. Categorical variables were presented as absolute frequencies and percentages, while Likert-scale items were presented using mean values and standard deviations.

The 17 Likert-scale items were grouped into four conceptual domains: perceived personal melanoma risk (5 items), melanoma-related concern (3 items), sun-protective behavior (5 items), and knowledge and attitudes toward melanoma (4 items). Each item was scored from 1 to 5, with higher values representing a higher level of the respective construct. For each participant, a composite score for each domain was calculated as the arithmetic mean of the corresponding item scores, resulting in domain scores ranging from 1 to 5. Higher domain scores therefore indicated greater perceived melanoma risk, greater melanoma-related concern, more frequent sun-protective behavior, or greater melanoma-related knowledge and more favorable attitudes, respectively. The categorical items describing demographic characteristics, melanoma history, risk factors, and other behaviors were analyzed descriptively and were not included in the calculation of the four Likert-scale composite domain scores. The internal consistency and reliability of each domain were assessed using Cronbach’s alpha coefficient, a widely used measure of internal consistency for multi-item scales [28,29].

Differences in domain scores according to gender and previous melanoma diagnosis were examined using independent samples *t*-tests. One respondent had no available responses to any of the 17 Likert-scale items; therefore, composite domain scores could not be calculated for this respondent. This case was retained in descriptive analyses for variables with available data but was excluded from analyses involving the Likert-scale domain scores. Effect sizes for between-group differences were calculated using Cohen’s d and are reported for all independent-samples *t*-tests. Absolute Cohen’s d values of approximately 0.20, 0.50, and 0.80 were interpreted as small, medium, and large effects, respectively. For variables with more than two categories, including age group, education level, and family history of melanoma, one-way ANOVA was applied. When the assumption of homogeneity of variances was violated, Welch’s robust test was interpreted. Tukey HSD or Games–Howell post hoc tests were used to identify specific pairwise differences between groups, depending on whether the assumption of equal variances was met.

Associations between the four composite domain scores were assessed using Spearman’s correlation coefficient. Finally, a general linear model was applied to identify factors associated with sun-protective behavior. The sun-protective behavior score was used as the dependent variable, while gender, age group, education level, previous melanoma diagnosis, and family history of melanoma were included as categorical predictors. Personal melanoma risk, melanoma-related concern, and knowledge and attitudes scores were included as covariates. Statistical significance was set at *p* < 0.05.

## 3. Results

The analysis of the results began with a descriptive overview of the respondents’ socio-demographic and clinical characteristics, followed by the assessment of Likert-scale items, composite domain scores, group differences, correlations, and predictors of sun-protective behavior.

Table 1 presents the socio-demographic characteristics of the respondents, including gender, age group, education level, employment status, previous melanoma diagnosis, and family history of melanoma.

The study included 333 respondents. Women were slightly more represented than men, accounting for 55.3% of the sample, while men accounted for 44.7%. Regarding age structure, the largest proportions of respondents were aged 66 years or older (42.3%) and 46–65 years (41.7%), whereas 15.9% were aged 45 years or younger. Most respondents had completed secondary school (42.9%) or college/university education (37.8%). A previous melanoma diagnosis was reported by 61.6% of respondents, while 15.9% reported a family history of melanoma.

Table 2 presents descriptive statistics for individual Likert-scale items assessing perceived personal melanoma risk, melanoma-related concern, sun-protective behavior, and knowledge and attitudes toward melanoma.

The descriptive analysis of Likert-scale items showed that respondents generally expressed high levels of melanoma-related concern, sun-protective behavior, and knowledge and attitudes. The highest mean values were observed for the statements related to the importance of early melanoma detection (Mean = 4.52, SD = 0.89), regular dermatological examinations even in the absence of symptoms (Mean = 4.49, SD = 0.92), and awareness that changes in the colour or shape of moles may indicate melanoma (Mean = 4.39, SD = 0.92). Among sun-protective behaviors, the highest mean value was recorded for avoiding tanning beds due to perceived danger (Mean = 4.34, SD = 1.03). Personal melanoma risk items had lower mean values, ranging from 2.94 to 3.23, indicating a moderate level of perceived personal risk among respondents.

Based on the previously presented Likert-scale items, four composite domain scores were calculated to summarize the main dimensions of the questionnaire: perceived personal melanoma risk, melanoma-related concern, sun-protective behavior, and knowledge and attitudes toward melanoma. Each score was calculated as the mean value of the corresponding items within the domain, with higher values indicating a higher level of the measured construct. Table 3 presents the descriptive statistics of these domain scores.

The highest mean score was observed for knowledge and attitudes toward melanoma (Mean = 4.40, SD = 0.82), indicating a generally high level of awareness regarding melanoma prevention, early detection, and the importance of dermatological examinations. Sun-protective behavior also had a high average score (Mean = 4.12, SD = 0.85), followed by melanoma-related concern (Mean = 4.04, SD = 0.88). The lowest mean score was recorded for perceived personal melanoma risk (Mean = 3.10, SD = 0.92), suggesting a moderate level of perceived personal risk among respondents. The reliability analysis showed acceptable to very good internal consistency across all Likert-scale domains. Cronbach’s alpha values ranged from 0.711 for melanoma-related concern to 0.874 for knowledge and attitudes. These results indicate that the items within each domain were sufficiently consistent and could be combined into composite scores for further analysis.

After the descriptive analysis of individual Likert-scale items and the calculation of composite domain scores, further analyses were conducted to examine whether these scores differed according to selected socio-demographic and clinical characteristics of the respondents. The analysis first focused on gender differences in perceived personal melanoma risk, melanoma-related concern, sun-protective behavior, and knowledge and attitudes toward melanoma. The results of the independent samples *t*-test comparing male and female respondents are presented in Table 4.

The results showed no statistically significant gender differences in perceived personal melanoma risk or knowledge and attitudes. However, statistically significant differences were found for melanoma-related concern and sun-protective behavior. Female respondents had higher mean scores for melanoma-related concern (Mean = 4.14, SD = 0.83) compared with male respondents (Mean = 3.93, SD = 0.94; *p* = 0.037, Cohen’s d = −0.235), as well as higher scores for sun-protective behavior (Mean = 4.23, SD = 0.76) compared with male respondents (Mean = 3.99, SD = 0.92; *p* = 0.011, Cohen’s d = −0.287). The corresponding effect sizes were small, indicating that although the gender differences were statistically significant, their magnitude was modest. Effect sizes for perceived personal melanoma risk (d = −0.124) and knowledge and attitudes (d = −0.036) were negligible to small and were not accompanied by statistically significant group differences.

In the next step, differences in the four composite Likert-scale domain scores were examined according to selected respondent characteristics. For variables with two categories, independent samples *t*-tests were used to compare mean scores between groups. In the case of previous melanoma diagnosis, respondents with and without a diagnosis were compared in terms of perceived personal melanoma risk, melanoma-related concern, sun-protective behavior, and knowledge and attitudes toward melanoma. When the assumption of equal variances was not met, Welch’s *t*-test results were interpreted. The results are presented in Table 5.

Respondents with a previous melanoma diagnosis had a significantly higher perceived personal melanoma risk score (Mean = 3.20, SD = 1.00) compared with respondents without a previous diagnosis (Mean = 2.94, SD = 0.77; *p* = 0.011, Cohen’s d = 0.277). The effect size was small, indicating a modest difference in perceived personal risk between the two groups. No statistically significant differences were observed for melanoma-related concern (*p* = 0.797, d = 0.027), sun-protective behavior (*p* = 0.721, d = 0.038), or knowledge and attitudes (*p* = 0.926, d = 0.010), with effect sizes for these comparisons being negligible. These findings indicate that a previous melanoma diagnosis was associated primarily with higher perceived personal risk, although the magnitude of this difference was small, while little to no difference was observed between the groups in concern, protective behavior, or melanoma-related knowledge and attitudes.

In addition to gender and previous melanoma diagnosis, differences in domain scores were also examined according to age group. Since age was categorized into three groups, one-way ANOVA was used to compare mean scores across age categories. When the assumption of homogeneity of variances was violated, Welch’s robust test was interpreted. Post hoc comparisons were used to identify specific differences between age groups. Table 6 presents differences in Likert-scale domain scores according to age group.

The results showed there were no statistically significant differences between age groups in the personal melanoma risk score (F = 0.909, *p* = 0.404). However, statistically significant age-related differences were observed for melanoma-related concern (Welch = 11.473, *p* < 0.001), sun-protective behavior (F = 4.594, *p* = 0.011), and knowledge and attitudes (F = 4.001, *p* = 0.019). Since the assumption of homogeneity of variances was violated for melanoma-related concern, Games–Howell post hoc test was used for this domain. The results showed that respondents aged 45 years or younger had significantly higher melanoma-related concern scores than respondents aged 46–65 years (*p* = 0.017) and those aged 66 years or older (*p* < 0.001), while the difference between the two older age groups was not statistically significant (*p* = 0.074). For sun-protective behavior, Tukey HSD post hoc test showed that respondents aged 45 years or younger had significantly higher scores than respondents aged 46–65 years (*p* = 0.027) and those aged 66 years or older (*p* = 0.010). Regarding knowledge and attitudes, Tukey HSD post hoc test showed that respondents aged 45 years or younger had significantly higher scores than respondents aged 66 years or older (*p* = 0.017), while other pairwise differences were not statistically significant.

In addition to gender, age group, and previous melanoma diagnosis, family history of melanoma was also examined as a potential factor associated with perceived risk, concern, protective behavior, and knowledge and attitudes. Table 7 presents differences in Likert-scale domain scores according to family history of melanoma. Since family history was categorized into three groups, one-way ANOVA was used to compare mean scores across groups. When the assumption of homogeneity of variances was violated, Welch’s robust test was interpreted.

There were no statistically significant differences between respondents according to family history of melanoma in the personal melanoma risk score (Welch = 2.515, *p* = 0.085) or knowledge and attitudes score (F = 1.899, *p* = 0.151). A statistically significant difference was found for melanoma-related concern (F = 3.078, *p* = 0.047), with the lowest mean score observed among respondents who did not know whether someone in their family had melanoma. However, Tukey HSD post hoc comparisons did not identify statistically significant pairwise differences between specific groups. For sun-protective behaviour, Welch’s test indicated a statistically significant difference between groups (Welch = 3.680, *p* = 0.028). Games–Howell post hoc comparisons suggested a borderline difference between respondents without a family history of melanoma and those who did not know their family history (*p* = 0.050), with higher protective behavior scores among respondents without a family history.

Education level was further examined as a potential factor associated with perceived personal melanoma risk, melanoma-related concern, sun-protective behavior, and knowledge and attitudes (Table 8). Since education was categorized into four groups, one-way ANOVA was used to compare mean domain scores across education levels. As the assumption of homogeneity of variances was met for all domains, Tukey HSD post hoc test was applied for pairwise comparisons.

The results showed no statistically significant differences between education groups in personal melanoma risk (F = 1.265, *p* = 0.286), sun-protective behavior (F = 2.600, *p* = 0.052), or knowledge and attitudes (F = 2.051, *p* = 0.107). A statistically significant difference was found only for melanoma-related concern (F = 3.815, *p* = 0.010). Tukey HSD post hoc test showed that respondents with college/university education had significantly higher melanoma-related concern scores compared with respondents with primary school education (*p* = 0.021). Other pairwise differences were not statistically significant. Although mean sun-protective behavior scores differed descriptively across education groups, the overall difference did not reach statistical significance (*p* = 0.052). Post hoc comparisons also did not identify statistically significant differences between education groups.

Finally, correlations between the four composite Likert-scale domain scores were examined in order to determine whether perceived personal melanoma risk, melanoma-related concern, sun-protective behaviour, and knowledge and attitudes were mutually associated. Since the scores were based on Likert-scale items, Spearman’s correlation coefficient was used. The results are presented in Table 9.

Spearman’s correlation analysis showed statistically significant positive associations between all four domain scores. Perceived personal melanoma risk was weakly but significantly correlated with melanoma-related concern, sun-protective behaviour, and knowledge and attitudes. Stronger positive correlations were observed between melanoma-related concern and sun-protective behaviour (rho = 0.604, *p* < 0.001), as well as between concern and knowledge and attitudes (rho = 0.537, *p* < 0.001). Sun-protective behaviour was also positively correlated with knowledge and attitudes (rho = 0.587, *p* < 0.001). These findings suggest that respondents with higher melanoma-related knowledge and attitudes also reported greater concern and more frequent sun-protective behaviour.

To further examine factors associated with sun-protective behaviour, a general linear model was applied with the sun-protective behaviour score as the dependent variable. Gender, age group, education level, previous melanoma diagnosis, and family history of melanoma were included as categorical predictors, while personal melanoma risk, melanoma-related concern, and knowledge and attitudes scores were included as covariates. The results are presented in Table 10.

The overall model was statistically significant and explained 61.2% of the variance in sun-protective behaviour scores (R^2^ = 0.612; adjusted R^2^ = 0.597; *p* < 0.001). The strongest predictors of sun-protective behaviour were knowledge and attitudes (F = 76.156, *p* < 0.001, partial η^2^ = 0.203) and melanoma-related concern (F = 69.859, *p* < 0.001, partial η^2^ = 0.189). Age group was also a statistically significant predictor (F = 3.215, *p* = 0.042), although with a smaller effect size. Gender showed a borderline association with sun-protective behaviour (*p* = 0.065), while education level, previous melanoma diagnosis, family history of melanoma, and personal melanoma risk score were not statistically significant predictors. These findings indicate that better melanoma-related knowledge and attitudes, together with higher concern about melanoma, are the main factors associated with more frequent sun-protective behavior.

## 4. Discussion

The present study evaluated melanoma-related risk perception, concern, knowledge, attitudes, and sun-protective behaviour among respondents attending the Oncology Institute of Vojvodina. Overall, participants demonstrated high levels of melanoma-related knowledge and preventive behaviour, whereas perceived personal melanoma risk remained moderate. Knowledge and attitudes represented the highest-scoring domain, indicating good awareness of the importance of early detection, regular dermatological examinations, and recognition of suspicious changes in pigmented lesions. Previous studies have similarly shown that greater melanoma awareness is associated with engagement in preventive practices and willingness to seek medical advice when suspicious skin changes are detected [15,24].

Importantly, the comparatively lower perceived personal risk despite high melanoma-related knowledge suggests that general disease awareness does not necessarily correspond to a strong perception of individual susceptibility. This discrepancy has also been described in cancer prevention research, where individuals may recognize the seriousness of a disease while underestimating their own vulnerability [17,20]. These findings support the importance of personalized risk communication that addresses both melanoma-related knowledge and individual risk perception.

Gender differences observed in this study revealed that women reported significantly higher levels of melanoma-related concern and more frequent sun-protective behaviour compared with men. Similar findings have been consistently reported in the literature, where women generally demonstrate greater engagement in preventive health behaviours and are more likely to adopt protective measures such as sunscreen use, seeking shade, and regular skin examinations [11,16]. The absence of significant gender differences in knowledge suggests that behavioural differences may be driven primarily by attitudes and risk perception rather than by access to information alone.

Respondents with a previous melanoma diagnosis reported significantly higher perceived personal melanoma risk compared with those without a previous diagnosis. Interestingly, previous diagnosis was not associated with significantly higher knowledge, concern, or protective behaviour. This finding suggests that although personal experience with melanoma increases awareness of susceptibility, it does not necessarily result in substantial differences in preventive practices. One possible explanation is that respondents attending an oncology institution already represent a population with relatively high overall awareness and exposure to health education.

Age-related analyses demonstrated that younger respondents exhibited significantly higher levels of melanoma-related concern, sun-protective behaviour, and knowledge compared with older participants. These findings may reflect increased exposure of younger generations to contemporary health education campaigns, digital health information, and public awareness initiatives [5,11]. Conversely, older individuals may have accumulated sun-exposure habits during periods when public knowledge regarding ultraviolet radiation risks was less widespread. The observed age differences indicate that older populations may benefit from specifically tailored educational strategies.

The observed high levels of melanoma-related knowledge among respondents may partly reflect the characteristics of the study population. Unlike studies conducted in the general population, the present survey included patients attending a specialized oncology institution, many of whom had previous experience with melanoma diagnosis, treatment, or follow-up procedures. Such exposure may increase awareness of melanoma warning signs, risk factors, and preventive measures. Similar observations have been reported by Wu et al. [24], who found that melanoma patients and their family members generally demonstrated higher levels of melanoma-related knowledge compared with population-based samples. Nevertheless, despite this relatively high level of knowledge, perceived personal melanoma risk remained only moderate, suggesting that knowledge alone may not be sufficient to influence individual risk perception.

This discrepancy between knowledge and perceived personal risk is particularly important from a public health perspective. Previous studies have demonstrated that individuals often acknowledge the seriousness of melanoma while simultaneously underestimating their own susceptibility to the disease [17,19]. Such optimism bias may reduce motivation for regular skin self-examination and adherence to preventive recommendations. Therefore, contemporary melanoma prevention strategies increasingly emphasize personalized risk communication, which combines objective risk assessment with tailored educational interventions [16,20]. The present findings support this approach and suggest that future prevention programs in Serbia should focus not only on increasing knowledge but also on strengthening individual risk awareness.

Family history of melanoma was associated with greater concern and more frequent sun-protective behaviour. Participants who were uncertain about their family history consistently demonstrated lower scores, suggesting that awareness of familial risk may contribute to greater engagement in preventive activities. These findings emphasize the importance of discussing family medical history during routine clinical consultations and melanoma prevention programs.

Educational level was significantly associated with melanoma-related concern, with respondents possessing higher education reporting greater concern than those with primary education. Although mean knowledge and protective behavior scores were descriptively higher in some education groups, these differences did not reach statistical significance and should therefore be interpreted as descriptive only. Higher educational attainment is generally associated with greater health literacy and better engagement with preventive health information [16,24]. The findings are particularly relevant in the Serbian context, where melanoma mortality continues to increase despite advances in diagnosis and treatment [9,10]. While considerable progress has been achieved in therapeutic management, comparatively less attention has been directed toward population-level prevention and behavioural interventions. The availability of a culturally adapted questionnaire capable of assessing melanoma-related risk perception, concern, knowledge, and protective behaviour may facilitate future epidemiological studies, educational programs, and targeted prevention strategies. Furthermore, the instrument may provide a practical method for identifying subgroups requiring additional counselling and preventive support within routine clinical practice.

An important finding of the present study was the significant positive correlation between all evaluated domains. Particularly strong associations were observed between melanoma-related concern and sun-protective behaviour, as well as between knowledge and protective behaviour. These findings support theoretical models of health behaviour suggesting that individuals who possess greater knowledge and perceive higher levels of concern are more likely to adopt preventive actions [15,16,20]. The correlation analysis therefore provides additional evidence supporting the conceptual validity of the questionnaire domains.

The multivariable analysis further demonstrated that melanoma-related knowledge and attitudes, together with melanoma-related concern, were the strongest predictors of sun-protective behaviour. Both variables explained a substantial proportion of behavioural variability, whereas demographic characteristics and previous melanoma diagnosis contributed relatively little once psychological and cognitive factors were taken into account. These findings suggest that interventions aiming to improve melanoma prevention should prioritize increasing knowledge and strengthening personal engagement with melanoma risk rather than focusing exclusively on demographic risk groups [16,20,24]. The growing interest in comparative oncology and the biological similarities between human and animal melanoma further emphasize the importance of preventive strategies and early detection programs across species [25].

The reliability analysis demonstrated acceptable to very good internal consistency across all questionnaire domains, with Cronbach’s alpha values ranging from 0.711 to 0.874. According to commonly accepted criteria, Cronbach’s alpha values above 0.70 indicate satisfactory internal consistency, while values above 0.80 reflect good reliability of multi-item scales [28,29]. These values are comparable to those reported in similar questionnaire-based studies evaluating melanoma-related knowledge, attitudes, and preventive behaviours and support the internal coherence of the adapted Serbian instrument. The findings indicate satisfactory consistency among questionnaire items and support the use of the instrument for future clinical, epidemiological, and educational studies in Serbia.

The questionnaire demonstrated satisfactory psychometric characteristics and acceptable to very good internal consistency across all evaluated domains. Significant associations observed between theoretically related constructs, including melanoma-related concern, knowledge and attitudes, and sun-protective behaviour, further support the applicability of the instrument in this clinical population. These findings suggest that the questionnaire may be a useful tool for assessing melanoma-related perceptions and behaviours among Serbian patients. Nevertheless, further studies involving larger and more diverse populations are warranted to continue the evaluation of its measurement properties and broader applicability.

Several limitations should be acknowledged. First, the study was conducted in a single oncology institution and utilized a convenience sample, limiting the generalizability of the findings. Second, all data were self-reported and may therefore be affected by recall bias and social desirability bias. Finally, the cross-sectional design precludes conclusions regarding causal relationships between knowledge, concern, risk perception, and protective behaviour. An important limitation is that the factorial structure of the questionnaire was not examined in the present study. Although internal consistency was satisfactory across the four predefined domains, Cronbach’s alpha alone does not establish the underlying dimensional structure of an instrument. Therefore, the proposed domain structure should be considered preliminary. Future validation studies should include exploratory factor analysis (EFA) to examine the underlying factor structure and confirmatory factor analysis (CFA) in an independent sample to evaluate whether the proposed four-domain structure is supported by the data.

Despite these limitations, the study provides important insights into melanoma-related perceptions and behaviors within a Serbian clinical population and supports the applicability of the questionnaire as a reliable tool for future melanoma prevention research.

## 5. Conclusions

The developed questionnaire provides a potentially useful tool for assessing melanoma-related risk perception, concern, knowledge, attitudes, and sun-protective behavior among Serbian patients. Its application may support the identification of individuals who could benefit from targeted risk communication and preventive education. However, given the preliminary nature of the present evaluation, further validation in larger and more diverse populations is required, including assessment of the questionnaire’s factorial structure using exploratory and confirmatory factor analyses.

Future studies should evaluate the questionnaire in broader and more diverse populations and further examine its measurement properties and applicability in different clinical settings.

## Figures and Tables

**Table 1:** Socio-demographic characteristics of respondents.

Variable	Category	*n*	%
Gender	Male	149	44.7
	Female	184	55.3
Age group	≤45 years	53	15.9
	46–65 years	139	41.7
	≥66 years	141	42.3
Education level	Primary school	43	12.9
	Secondary school	143	42.9
	College/University	126	37.8
	Master/PhD	21	6.3
Employment status	Employed	130	39.0
	Unemployed	45	13.5
	Retired	155	46.5
	Student	3	0.9
Previous melanoma diagnosis	Yes	205	61.6
	No	128	38.4
Family history of melanoma	Yes	53	15.9
	No	192	57.5
	I do not know	88	26.4

**Table 2:** Descriptive statistics of Likert-scale items.

Domain	Item	Mean	SD
Personal melanoma risk	My skin burns easily in the sun.	3.20	1.12
	I have many moles on my body.	3.23	1.20
	I have moles of irregular shape or color.	2.94	1.22
	My skin freckles easily when exposed to the sun.	3.00	1.21
	I have had sunburn several times in the past.	3.05	1.26
Melanoma-related concern	I regularly check my skin for moles or changes.	3.92	1.12
	If I noticed a new skin change, I would probably consult a doctor within the next month.	4.19	1.02
	I am concerned about the risk of developing melanoma.	4.06	1.05
Sun-protective behavior	I try to avoid sun exposure between 10 a.m. and 4 p.m.	4.22	0.99
	When I am in the sun, I always seek shade.	4.17	1.00
	I regularly use sunscreen SPF 30+.	3.97	1.16
	I wear protective clothing.	3.95	1.15
	I consider tanning beds dangerous and therefore avoid them.	4.34	1.03
Knowledge and attitudes	Early detection of melanoma significantly increases the chances of cure.	4.52	0.89
	Changes in the color or shape of moles may indicate melanoma.	4.39	0.92
	People with darker skin can also develop melanoma.	4.20	1.05
	Regular dermatological examinations are important even when there are no symptoms.	4.49	0.92

**Table 3:** Descriptive statistics of Likert-scale domain scores.

Domain	Mean	SD	Cronbach’s Alpha	Number of Items
Personal melanoma risk	3.10	0.92	0.776	5
Melanoma-related concern	4.04	0.88	0.711	3
Sun-protective behavior	4.12	0.85	0.807	5
Knowledge and attitudes	4.40	0.82	0.874	4

**Table 4:** Gender differences in Likert-scale domain scores.

Domain	Male Mean ± SD	Female Mean ± SD	t	*p*-Value	Cohen’s d
Personal melanoma risk	3.03 ± 0.96	3.15 ± 0.90	−1.103	0.271	−0.124
Melanoma-related concern	3.93 ± 0.94	4.14 ± 0.83	−2.092	0.037	−0.235
Sun-protective behavior	3.99 ± 0.92	4.23 ± 0.76	−2.546	0.011	−0.287
Knowledge and attitudes	4.38 ± 0.89	4.41 ± 0.76	−0.317	0.751	−0.036

**Table 5:** Differences in Likert-scale domain scores according to previous melanoma diagnosis.

Domain	Previous Diagnosis: Yes Mean ± SD	Previous Diagnosis: No Mean ± SD	t	*p*-Value	Cohen’s d
Personal melanoma risk	3.20 ± 1.00	2.94 ± 0.77	2.566	0.011	0.277
Melanoma-related concern	4.05 ± 0.99	4.03 ± 0.68	0.258	0.797	0.027
Sun-protective behavior	4.13 ± 0.98	4.10 ± 0.60	0.358	0.721	0.038
Knowledge and attitudes	4.40 ± 0.94	4.39 ± 0.60	0.093	0.926	0.010

Note: One respondent without a previous melanoma diagnosis had no available responses to the 17 Likert-scale items and was therefore excluded from the domain-score comparisons. Accordingly, these analyses included 332 respondents (previous diagnosis: Yes, *n* = 205; No, *n* = 127).

**Table 6:** Differences in Likert-scale domain scores according to age group.

Domain	≤45 Years Mean ± SD	46–65 Years Mean ± SD	≥66 Years Mean ± SD	Test Statistic	*p*-Value
Personal melanoma risk	3.10 ± 0.90	3.17 ± 0.86	3.02 ± 0.99	F = 0.909	0.404
Melanoma-related concern	4.41 ± 0.62	4.09 ± 0.89	3.85 ± 0.92	Welch = 11.473	0.000
Sun-protective behavior	4.43 ± 0.69	4.08 ± 0.85	4.03 ± 0.88	F = 4.594	0.011
Knowledge and attitudes	4.63 ± 0.64	4.43 ± 0.81	4.27 ± 0.88	F = 4.001	0.019

**Table 7:** Differences in Likert-scale domain scores according to family history of melanoma.

Domain	Yes Mean ± SD	No Mean ± SD	I Do Not Know Mean ± SD	Test Statistic	*p*-Value
Personal melanoma risk	3.32 ± 0.85	3.08 ± 1.00	3.00 ± 0.78	Welch = 2.515	0.085
Melanoma-related concern	4.16 ± 0.78	4.11 ± 0.97	3.85 ± 0.72	F = 3.078	0.047
Sun-protective behavior	4.22 ± 0.81	4.17 ± 0.94	3.94 ± 0.64	Welch = 3.680	0.028
Knowledge and attitudes	4.51 ± 0.75	4.43 ± 0.87	4.26 ± 0.75	F = 1.899	0.151

**Table 8:** Differences in Likert-scale domain scores according to education level.

Domain	Primary School Mean ± SD	Secondary School Mean ± SD	College/University Mean ± SD	Master/PhD Mean ± SD	F	*p*-Value
Personal melanoma risk	3.17 ± 0.92	3.08 ± 0.95	3.03 ± 0.88	3.44 ± 1.01	1.265	0.286
Melanoma-related concern	3.74 ± 0.87	3.96 ± 0.94	4.20 ± 0.80	4.25 ± 0.85	3.815	0.010
Sun-protective behavior	3.98 ± 0.86	4.00 ± 0.89	4.24 ± 0.77	4.36 ± 0.85	2.600	0.052
Knowledge and attitudes	4.22 ± 0.83	4.32 ± 0.91	4.53 ± 0.69	4.48 ± 0.88	2.051	0.107

**Table 9:** Correlations between Likert-scale domain scores.

Variables	Spearman’s Rho	*p*-Value
Personal melanoma risk—Melanoma-related concern	0.263	0.000
Personal melanoma risk—Sun-protective behavior	0.154	0.006
Personal melanoma risk—Knowledge and attitudes	0.120	0.034
Melanoma-related concern—Sun-protective behavior	0.604	0.000
Melanoma-related concern—Knowledge and attitudes	0.537	0.000
Sun-protective behavior—Knowledge and attitudes	0.587	0.000

**Table 10:** Predictors of sun-protective behavior.

Predictor	F	*p*-Value	Partial Eta Squared
Gender	3.439	0.065	0.011
Age group	3.215	0.042	0.021
Education level	0.591	0.621	0.006
Previous melanoma diagnosis	0.010	0.920	0.000
Family history of melanoma	0.666	0.515	0.004
Personal melanoma risk score	0.364	0.547	0.001
Melanoma-related concern score	69.859	0.000	0.189
Knowledge and attitudes score	76.156	0.000	0.203

## Data Availability

The data presented in this study are available from the corresponding author upon reasonable request. The data are not publicly available due to privacy and ethical restrictions.

## Abbreviations

The following abbreviations are used in this manuscript:

CM	Cutaneous Melanoma
SPF	Sun Protection Factor
SD	Standard deviation
ANOVA	Analysis of Variance
HSD	Honestly Significant Difference
R^2^	Coefficient of Determination
